# Survival and recovery: 24-month outcomes for critically Ill COVID-19 patients receiving ECMO

**DOI:** 10.3389/fmed.2025.1731333

**Published:** 2026-01-12

**Authors:** Zsuzsanna Ulakcsai, Zsófia Dohy, Liliána Szabó, Dorottya Balla, Csongor Meskó, Bálint Lakatos, Daniele Fontanini Mariastefano, Dorottya Fésü, Zsófia Ocsovszky, József Otohal, Blanka Ehrenberger, Zsófia Szabó, Tamás Szabó, Endre Németh, Veronika Müller, György Nagy, Hajnalka Vágó, Béla Merkely

**Affiliations:** 1 Heart and Vascular Center, Semmelweis University, Budapest, Hungary; 2 Department of Pulmonology, Semmelweis University, Budapest, Hungary; 3 Department of Laboratory Medicine, Semmelweis University, Budapest, Hungary; 4 Department of Rheumatology and Immunology, Semmelweis University, Budapest, Hungary; 5 Department of Genetics, Cell and Immunobiology, Semmelweis University, Budapest, Hungary; 6 Department of Sports Medicine, Semmelweis University, Budapest, Hungary

**Keywords:** COVID-19, ECMO, immune response, long-term follow-up, multidisciplinary approach, quality of life, SARS-CoV-2

## Abstract

**Background:**

Veno-venous extracorporeal membrane oxygenation (V-V ECMO) was used for patients with severe COVID-19 pneumonia. The aim of our study was to assess the long-term outcomes and quality of life of the surviving patients.

**Methods:**

This single-center observational study was performed among patients who were discharged after V-V ECMO treatment with COVID-19 pneumonia. During the 2-year follow-up period, different organ functions and quality of life parameters were evaluated three times after discharge. As a control group, SARS-CoV-2 infection positive patients were included.

**Results:**

Thirty-five patients underwent V-V ECMO treatment, of whom 11 patients survived. The study population consists of 9 patients for the follow-up (2 patients did not consent to follow-up examinations). On lung CT, the occurrence of residual fibrotic banding was 22% at discharge, and increased to 89% at 24th month follow-up, while lung function tests were normal. On echocardiography, patients had a mildly elevated right ventricle end-diastolic diameter at the post discharge period, which normalized during follow-up (baseline: 37.6 ± 2.9 mm, follow-up: 35.6 ± 2.2 mm, *p* < 0.05). In the ECMO group both humoral and cellular immune responses remained elevated (Quantiferon Ag1 *p* = 0.005, Quantiferon Ag2 *p* = 0.0059, Quantiferon Ag3 *p* = 0.0012, IgG Roche *p* = 0.0037).

Among the psychological factors, significant correlation was observed between ECMO duration and symptoms of depression (*r* = 0.727; *p* < 0.05), anxiety (*r* = 0.848; *p* < 0.01), and posttraumatic stress (*r* = 0.834; *p* < 0.01), furthermore, a negative correlation with positive affectivity (*r* = −0.868; *p* < 0.01) presented itself. SF-36 scores improved significantly from the 6-month to the 24-month follow-up (median 50% vs. 80%, *p* < 0.01). The mean body mass index increased from 29 to 35 (kg/m^2^) and physical activity decreased. The Epworth Sleepiness Scale showed higher normal daytime sleepiness values and elevated Fatigue Severity Scale scores.

**Conclusion:**

At the 2-year follow-up after discharge from the V-V ECMO treatment, permanent cardio muscular and structural pulmonary damage had been anticipated. However, on one hand, good cardiac and pulmonary function and robust sustained immune response were measured. On the other hand, unforeseen long-term complications arose, such as sleepiness, reduced physical activity, acute depression, and consequently, a deteriorated health related quality of life. These findings highlight the necessity of long-term, structured controlled rehabilitation programs for this patient population.

## Graphical Abstract

**Figure:**
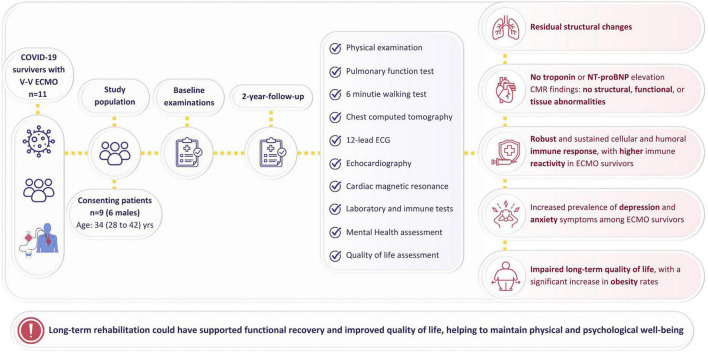

## 1 Introduction

SARS-CoV-2 infection and the resulting COVID-19 pandemic have had profound effects on global morbidity and mortality, leading to widespread illness, long-term health complications, and millions of deaths, while placing unprecedented strain on healthcare systems and societies worldwide. The intensive care units faced extraordinary challenges. The emergence and spread of the Delta variant (SARS-CoV-2 virus Delta B.1.617.2) resulted in a surge of infections and record numbers of hospitalizations, particularly among the unvaccinated population. Approximately 5–7% of patients with COVID-19 caused by the Delta variant have developed critical lung injury and acute respiratory distress syndrome (ARDS). In severe ARDS, when mechanical ventilation is unable to maintain adequate oxygenation and/or CO_2_ elimination, venovenous extracorporeal membrane oxygenation (V-V ECMO) may be considered to support gas exchange and minimize ventilator-induced lung injury (1, 2). At the peak of the pandemic, V-V ECMO support was implemented by tertiary cardiovascular centers to treat critically ill COVID-19 patients (3).

The determinants of in-hospital mortality and the selection criteria were reported in our previous study (4). However, limited data are available on the long-term outcomes after discharge from the hospital following ECMO support (5–8). During our therapeutic efforts, the most frequently raised question was whether survivors who had received ECMO support would regain an acceptable quality of life. During the follow-up period, the most common clinical complaints of our patients were tachycardia, effort dyspnea, fatigue and reduced physical capacity. We hypothesized that these symptoms could result from organ damage of cardiac or pulmonary origin caused by severe COVID-19. Our secondary hypothesis was that survivors of critical illness remain at increased risk of morbidity even after hospital discharge. These long-term consequences, widely referred to as post-intensive care syndrome (PICS), include mental, physical/organic, and neurocognitive deficits that negatively affect recovery, rehabilitation, and further quality of life (9). The aim of our present study was to investigate the long-term outcome of patients receiving ECMO support for severe COVID-19 disease-causing ARDS. We investigated in detail the cardiac, pulmonary, immunological, and mental status and quality of life of the patients during a follow-up period of at least 24 months.

## 2 Materials and methods

### 2.1 Study population

We conducted a prospective single-center observational study at the Heart and Vascular Center of Semmelweis University that included patients who survived SARS-CoV-2 infection with VV-ECMO support, between March 1 and December 31 2021. All of them were infected with the Delta B.1.6.17.2 variant of the SARS-COV-2 virus. The inclusion criteria were as follows: (1) > 18 years of age, (2) laboratory-confirmed SARS-CoV-2 infection (by real-time PCR), (3) V-V ECMO support due to refractory COVID-19-related ARDS, and (4) discharge from the ICU after successful treatment. Patients who did not consent to follow-up were excluded from the study. All the patients included in our study were classified as severely ill (D10) according to the World Health Organization (WHO) criteria as of 27th May 2020 (10). The ICU care protocol was described in our previous publication (4). In brief, all patients had chest CT, echocardiography and laboratory examinations, including immunological measurements during acute infection. We followed the updated recommendations of the Extracorporeal Life Support Organization COVID-19 Interim Guidelines (11).

Ethical approval was obtained from the National Public Health Center under the ethical standards laid out in the 1964 Declaration of Helsinki and its later amendments (IV/2568-1/2021/EKU). All participants or their legal guardians provided written informed consent for participation in the analysis. It was not possible to involve patients or the public in the process of designing, conducting, reporting, or disseminating our research.

### 2.2 Follow-up

Patients were followed for at least 24 months after discharge from the ICU. Nine of the eleven surviving patients participated in the long-term follow-up studies. The final evaluations were conducted between 24 and 36 months after the patients’ diagnosis of COVID-19 and included detailed pulmonary, cardiac, immune and psychological evaluations. Certain tests were repeated during the follow-up period, depending on the patients’ clinical condition.

#### 2.2.1 Pulmonology

All patients underwent a baseline noncontrast chest CT scan upon admission to the emergency department as part of the routine imaging protocol for patients hospitalized with severe pneumonia during the COVID-19 pandemic. The baseline and follow-up CT images were interpreted by a board-certified radiologist specializing in cardiothoracic imaging (DMF). CT evaluation was performed using syngo.via software (Siemens Healthineers AG, Forchheim, Germany).

Patients underwent physical examination and underwent the 6-minute walk test (6MWT) along with pulmonary function tests (PFTs) in accordance with ATS/ERS guidelines (12); these tests included measurements of forced vital capacity (FVC), forced expiratory volume in the first second (FEV1), and FEV1/FVC. The diffusing capacity of the lungs for carbon monoxide (DLCO) was determined through the single-breath CO method, and the carbon monoxide transfer coefficient (KLCO) was calculated (PDD-301/s, Piston, Budapest, Hungary) (13). Considering the severity of the pandemic and the workload at our hospital, we were only able to complete the control respiratory function tests in the 24th month of follow-up.

#### 2.2.2 Cardiology

During the follow-up, all ECMO patients underwent a standard 12-lead electrocardiogram (ECG), laboratory test with N-terminal pro-B-type natriuretic peptide (NT-proBNP) and high-sensitivity (hs) troponin T measurements, echocardiography and cardiac magnetic resonance (MR) examination.

Baseline echocardiography was performed within 24 h after ECMO implantation. Conventional echocardiographic exams were performed using a dedicated cardiac ultrasound system (GE Vivid E95, equipped with a 4Vc-D matrix-array transducer; GE Healthcare, Horten, Norway). A standard acquisition protocol consisting of loops from parasternal, apical, and subxiphoid views was implemented in accordance with the current guidelines (14).

Cardiac MR images were acquired on a 1.5 T MR scanner (Magnetom Aera; Siemens Healthcare, Erlangen, Germany). The cardiac MR protocol included cine movies, T2 mapping, T1 mapping, and late gadolinium enhancement (LGE) imaging. Control group examinations were performed without contrast agent. Cardiac MR images were analyzed by a blinded observer using Medis Suite Software (Medis Medical Imaging Software, The Netherlands). Left (LV) and right ventricular (RV) volumes, function, and mass were calculated. Myocardial native T1 and T2 relaxation times were measured in basal and midventricular SA slices. Global strain, including the LV global longitudinal (GLS), circumferential strain (GCS), RV longitudinal, and free wall strain, was measured.

#### 2.2.3 Immunology

Immunological measurements were performed on venous peripheral blood samples at 6, 12, 24, and 30 months after COVID-19. The humoral immune response was assessed by a specific antibody assay (spec IgG Roche), and the SARS-CoV-2-specific T-cell immune response was assessed by a quantification assay (QF-Ag1, QF-Ag2, QF-Ag3). The detailed methodology used for immunological and marker measurements was described in our previous article (4).

#### 2.2.4 Psychology

To evaluate the participants’ mental health status and quality of life, validated questionnaires were used. To measure depressive symptoms, the Beck Depression Inventory was used (15). Trait anxiety was evaluated by the Spielberger Trait Anxiety Inventory (15). Posttraumatic stress syndrome symptoms were assessed with the Posttraumatic Diagnostic Scale (15). The participants’ affectivity (positive or negative) was examined with the Positive and Negative Affectivity Schedule (16). Mental and physical quality of life was measured by the 36-Item Short Form Health Survey (17). The 1-item Life Satisfaction Scale was used to assess life satisfaction (18).

#### 2.2.5 Quality of life

Data pertaining to the general quality of life were recorded, including the ED-5Q-3 L score and the visual analog scale-VAS score (19). Questionnaires, namely, the Pittsburgh Sleep Quality Index (PSQI) (20), the Epworth Sleepiness Scale (ESS) (21), and the Fatigue Severity Scale (22), were administered to assess sleep habits and sleep-related symptoms.

### 2.3 Control groups

An age- and sex-matched control group of 10 individuals were selected for the immunological analyses. The control group comprised patients who had been infected with the SARS-CoV-2 at the same time as the ECMO-treated cohort (all infected with the Delta B.1.6.17.2 variant of the SARS-COV-2 virus) and who were also unvaccinated at the time of infection. The control group did not require hospital admission, drug therapy or supplemental oxygen therapy and recovered at home. Patients were classified as having mild symptomatic disease if they met the WHO case definition for COVID-19 without evidence of viral pneumonia or hypoxia, according to the criteria issued on 27th May 2020 (10). Members of the control group received the same vaccination as the ECMO-treated group, administered at the same time—approximately 6–7 months after infection. RNA-based vaccines, such as BNT162b2, were administered.

To evaluate the cardiac MR data, we selected a healthy control group of 15 individuals of our clinic’s database who had MR scans taken before the COVID-19 pandemic. The control group was matched for age and sex to the ECMO patients.

### 2.4 Statistical analysis

Statistical analysis was carried out using MedCalc software (version 17.9.5), R (version v4.3.0) and GraphPad. Correlation analysis was performed using Spearman’s rho test. Measurements at different time points, originating from the same individual, were compared using the paired-sample version of Wilcoxon signed rank test. Comparisons between two groups at the same time points were made using the Mann–Whitney U test. To reconstruct the dynamics of changes in the immune response, the median values were calculated for each cohort at each time point. Confidence intervals around these medians were calculated using the DescTools package and the bootstrap method. Differences were considered to be statistically significant at *p* < 0.05.

## 3 Results

### 3.1 Patient characteristics

A total of 35 critically ill patients with COVID-19 received VV-ECMO support in the ICU of the Heart and Vascular Center between 1 March and 31 December 2021, 11 of whom survived the disease. The study included only 9 of these survivors, and two patients declined participation in the follow-up. The mean follow-up time was 32 ± 5 months. The patients’ characteristics at the time of acute COVID-19 disease are summarized in Table 1.

**TABLE 1:** Patients’ baseline characteristics.

Variable	Occurence	
Male (%)	66.7% (6/9)	
Female (%)	33.3% (3/9)	
Age (years) median (IQR)	34 (28–42)	
Body mass index (kg/m^2^) median (IQR)	29.2 (27–30.1)	
Hospitalization length (days) median (IQR)	63 (48–78)	
ECMO therapy length (days) median (IQR)	12 (11–23)	
Rehabilitation length (days) median (IQR)	30 (21–67)	
**Patients’ history**	**Frequency**	
Obesity	22.2% (2/9)	
Hypertension	22.2% (2/9)	
Diabetes	22.2% (2/9)	
Smoking	0% (0/9)	
Atrial fibrillation	0% (0/9)	
Ischemic heart disease	11.1% (1/9)	
Chronic obstructive pulmonary disease	0% (0/9)	
**Laboratory parameters**	**Result**	**Reference range**
Baseline hs-troponin T (ng/L) median (IQR)	11 (6.9–21)	0–14
Peak hs-troponin T (ng/L) median (IQR)	20 (14.5–68.3)	0–14
Baseline NT-proBNP (ng/L) median (IQR)	235 (149–299)	0–125
Peak NT-proBNP (ng/L) median (IQR)	304 (227–629)	0–125
**Cardiac echo parameters**	**Result**	**Reference range**
LVEDD (mm) median (IQR)	47 (44–51)	42–58
LVEF (%) median (IQR)	58 (55–61)	53–73
RVEDD (mm) median (IQR)	37 (36–40)	25–41
TAPSE (mm) median (IQR)	23 (22–25)	>17
RVFAC (%) median (IQR)	40 (37–43)	>35

### 3.2 Pulmonology

PFTs after 2 years (Time since infection 968 +/– 155 days) showed that three patients had mild restrictive ventilatory dysfunction, two patients had mild obstructive ventilatory dysfunction, and one patient displayed a combined respiratory ventilatory pattern. In three patients, the lung function values were normal. The results of respiratory function tests are summarized in Table 2.

**TABLE 2:** The PFT results of COVID-19 ECMO patients at their last post-COVID visit.

Pulmonary function parameters	COVID-19 ECMO group (*N* = 9) mean ± standard deviation	
Variable	Result	Reference range
FEV1 (L)	3.3 ± 0.6	3–4
FEV1 (%)	82.7 ± 10.8	> 80
FVC (L)	3.9 ± 0.7	3–5
FVC (%)	81.3 ± 10.9	>80
FEV1/FVC (%)	82.7 ± 5.6	>80
TLC (L)	6.0 ± 2.1	5–7
TLC (%)	90.1 ± 16.9	>80
RV (L)	1.84 ± 0.73	1.0–1.5
RV (%)	104.4 ± 28.7	80–120
DLco (mmol/min/kPa)	12.7 ± 4.9	20–35
DLco (%)	127.7 ± 27.5	75–140
KLco (mmol/min/kPa)	2.2 ± 0.8	1.8–2.5
KLco (%)	132.1 ± 19.0	> 80
6MWT (m)	506 ± 60	600–800 ± 50
HR at start (/min)	83 ± 5	60–85
HR at end (/min)	117 ± 14	110–150
SpO_2_ at start (%)	98 ± 1	>96
SpO_2_ at end (%)	94 ± 3	>94
Desaturation > 5% (N %)	2 (22)	>5

FEV1, forced expiratory volume in the first second; FVC, forced vital capacity; DLco, diffusing capacity of the lungs for carbon monoxide; KLco, carbon monoxide transfer coefficient; 6MWT, 6-minute walk test; HR, heart rate.

**FIGURE 1:**
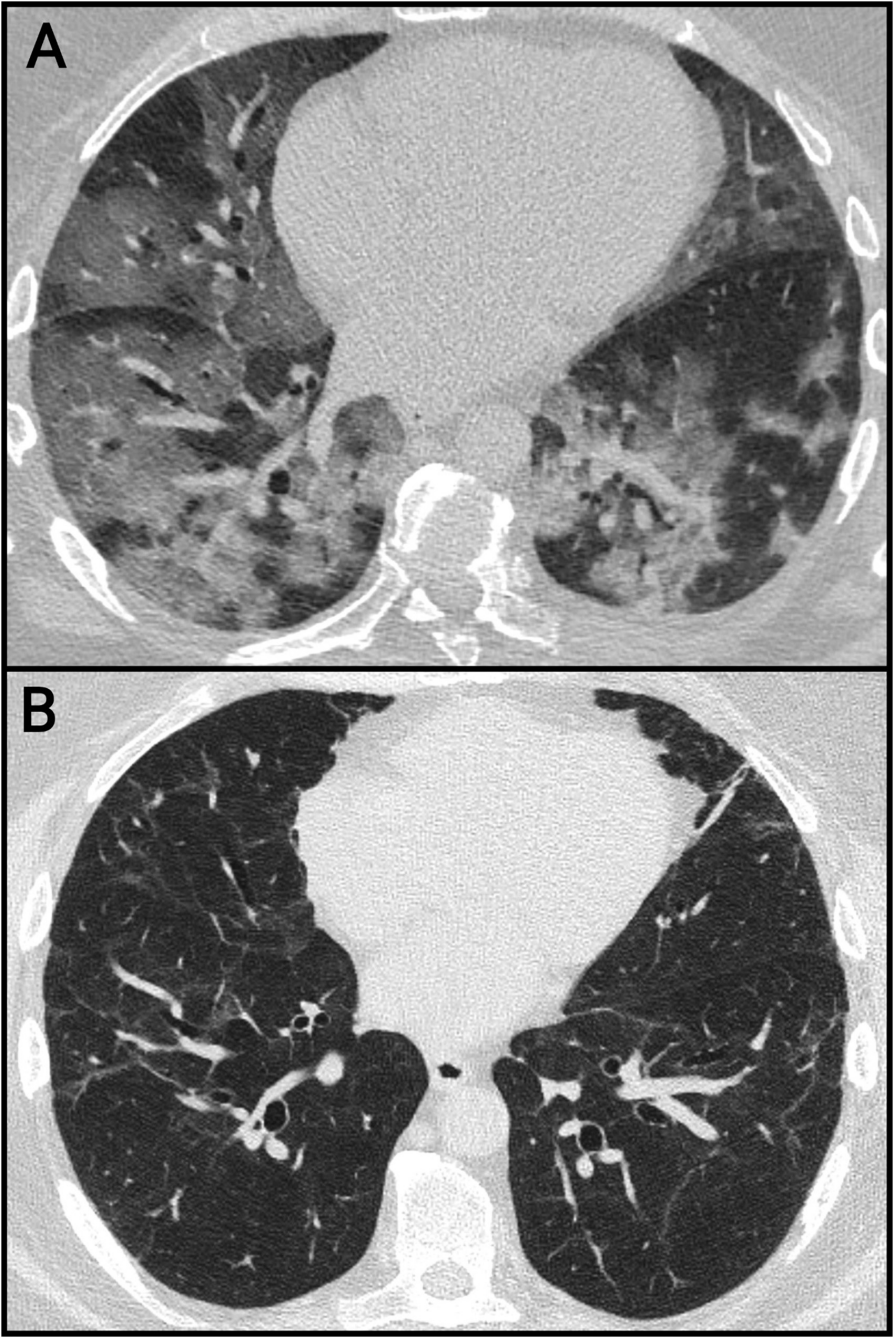
Lung changes on baseline and follow-up chest CT images. The baseline scan **(A)** shows extensive patchy and confluent ground-glass opacities. The 24-month follow-up scan **(B)** shows residual banding and mild traction bronchiectasis.

The CT findings of the baseline scans and follow-up examinations are shown in Table 3. On admission, 6 patients (66.7%) presented with severe infection; in these patients, more than 75% of the lungs were affected. Ground-glass opacities (GGOs) and consolidations were initially present in 8 patients (89% of patients) (Figure 1). The presence of GGOs regressed to 22% in follow-up studies. The consolidation completely resolved at the later follow-ups. Reactive lymphadenomegaly was identified in four patients (45%) at baseline but was absent on subsequent scans. The presence of residual fibrotic banding, initially noted in two patients (22%), increased significantly to 8 patients (89%) at both the 6- and 24-month follow-up examinations.

**TABLE 3:** CT findings at baseline and during follow-up.

CT Morphology	Baseline scan	6-months follow-up	24-months follow-up
GGO (n, %)	8 (89%)	2 (22%)	2 (22%)
Consolidations (n, %)	8 (89%)	0 (0%)	0 (0%)
Lymphadenomegaly (n, %)	4 (45%)	0 (0%)	0 (0%)
Traction bronchiectasis (n, %)	1 (11%)	3 (33%)	2 (22%)
Residual banding (n, %)	2 (22%)	8 (89%)	8 (89%)

GGO, ground-glass opacity.

### 3.3 Cardiology

During severe COVID-19, 7 patients had mildly elevated hs-troponin T levels, and two patients had no hs-troponin T elevation. The NT-proBNP level was significantly elevated in one patient, and the remaining patients had mildly elevated NT-proBNP levels (Table 1). During follow-up, all patients had normal hs-troponin T and NT-proBNP levels. On follow-up, 12-lead ECGs showed no clinically relevant abnormalities; all patients had sinus rhythm with a mean heart rate of 71 ± 10 beats/min, one patient had right bundle branch block, and another patient had incomplete right bundle branch block.

On echocardiography, patients had a mildly elevated RV end-diastolic diameter (EDD) in the acute period, which normalized during follow-up (baseline RVEDD: 37.6 ± 2.9 mm, follow-up RVEDD 35.6 ± 2.2, *p* < 0.05). LV and RV function were within normal ranges throughout, and no significant valvular abnormalities were observed (Table 1). None of the patients had more than a trace amount of tricuspid regurgitation, indicating that the pulmonary arterial peak systolic pressure was accurate.

All patients after COVID-19 diagnosis and ECMO therapy had normal cardiac MR parameters, including ventricular volume, function, strain parameters, and mapping values, at both the sixth and 24th month follow-up examinations. Only one patient with a history of myocardial infarction prior to COVID-19 disease had detectable pathognomic contrast enhancement with a pattern consistent with the history of myocardial infarction. No patients showed contrast enhancement or mapping abnormalities indicative of myocardial injury related to COVID-19. A comparison of the COVID-19 ECMO patients and the healthy controls revealed that the COVID-19 patients had lower LV and RV volumes (Table 4).

**TABLE 4:** Cardiac MR parameters of healthy controls and patients after COVID-19 diagnosis and ECMO support.

Variable	Control group		COVID-19 ECMO group		
	Median	IQR	Median	IQR	*P*
Age (years)	37.3	33.5–43.2	37.7	32.3–47.5	0.83
LVEDVi (mL/m^2^)	87	81–96.25	72	66.75–84.75	0.02
LVESVi (mL/m^2^)	34	33–38.75	27	25.5–32.25	0.01
LVSVi (mL/m^2^)	53	49–54.75	46	41–53.25	0.11
LVEF (%)	60	56.25–62.5	63	61.5–64.25	0.10
LVMi (g/m^2^)	49	45–52	42	39–54	0.42
LV GCS (%)	−29.8	−31.4 to −28.4	−33	−36.5 to −29.6	0.17
LV GLS (%)	−20.1	−24.5 to −19.4	−22	−22.9 to −20.7	0.70
RVEDVi (mL/m^2^)	89	85.5–99	80	70.75–87.25	0.04
RVESVi (mL/m^2^)	41	36.5–42.75	35	30.75–38.5	0.02
RVSVi (mL/m^2^)	48	47–54.25	46	40–51.75	0.36
RVEF (%)	55	53.25–58	57	53.75–60	0.51
RV free wall LS (%)	−34.3	−39.5 to −33.9	−35.5	−37.1 to −31.8	0.88
RV GLS (%)	−26.7	−30.8 to −25.7	−28.3	−31.1 to −25.8	0.64
T1 basis (ms)	968	956–996	972	962–984	0.61
T1 mid (ms)	960	927–981	951	937–976	0.52
T2 basis (ms)	45	44–45	44	44–46	0.78
T2 mid (ms)	46	45–47	44	43–46	0.06

LVEDVi, Left Ventricular End-Diastolic Volume index; LVESVi, Left Ventricular End-systolic Volume index; LVSVi, Left Ventricular Stroke Volume index; LVEFi, Left Ventricular Ejection Fraction index; LVMi, Left Ventricular Mass index; LV GCS, Left Ventricular Global Contractile Strain; LV GLS, Left Ventricular Global Longitudinal Strain; R, Right.

### 3.4 Immunology

SARS-CoV-2-specific antibodies and T-cell responses were detected in blood samples from all individuals in the ECMO group and the control group at 6 months after COVID-19. There was no significant difference in the expression of humoral or cellular immune response markers between the two groups. The control group had a significantly greater humoral (IgG) immune response.

**FIGURE 2:**
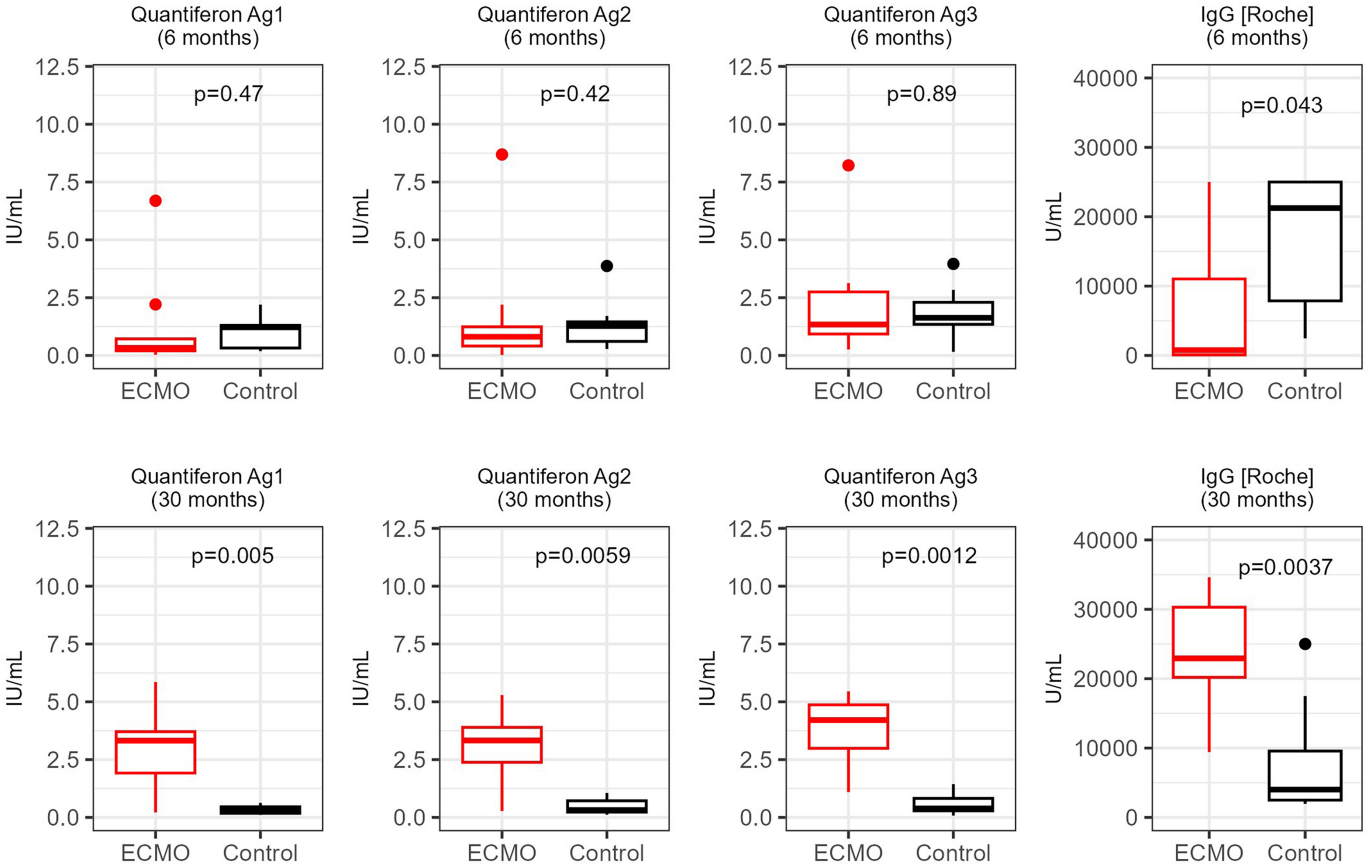
Differences in immune parameters between severe and less patients with severe COVID-19 at the initial and final follow-ups. Both cellular (Quantiferon) and humoral (IgG) immune responses against SARS-CoV-2 were measured in patients requiring ECMO treatment and in a control group of patients with less severe COVID-19. Immune reactivity at the first follow-up visit (6 months), as well as at the last available visit (30 months), was compared between the two groups using Wilcoxon signed rank test. The raw *p* values are shown before any correction for multiple hypotheses.

**FIGURE 3:**
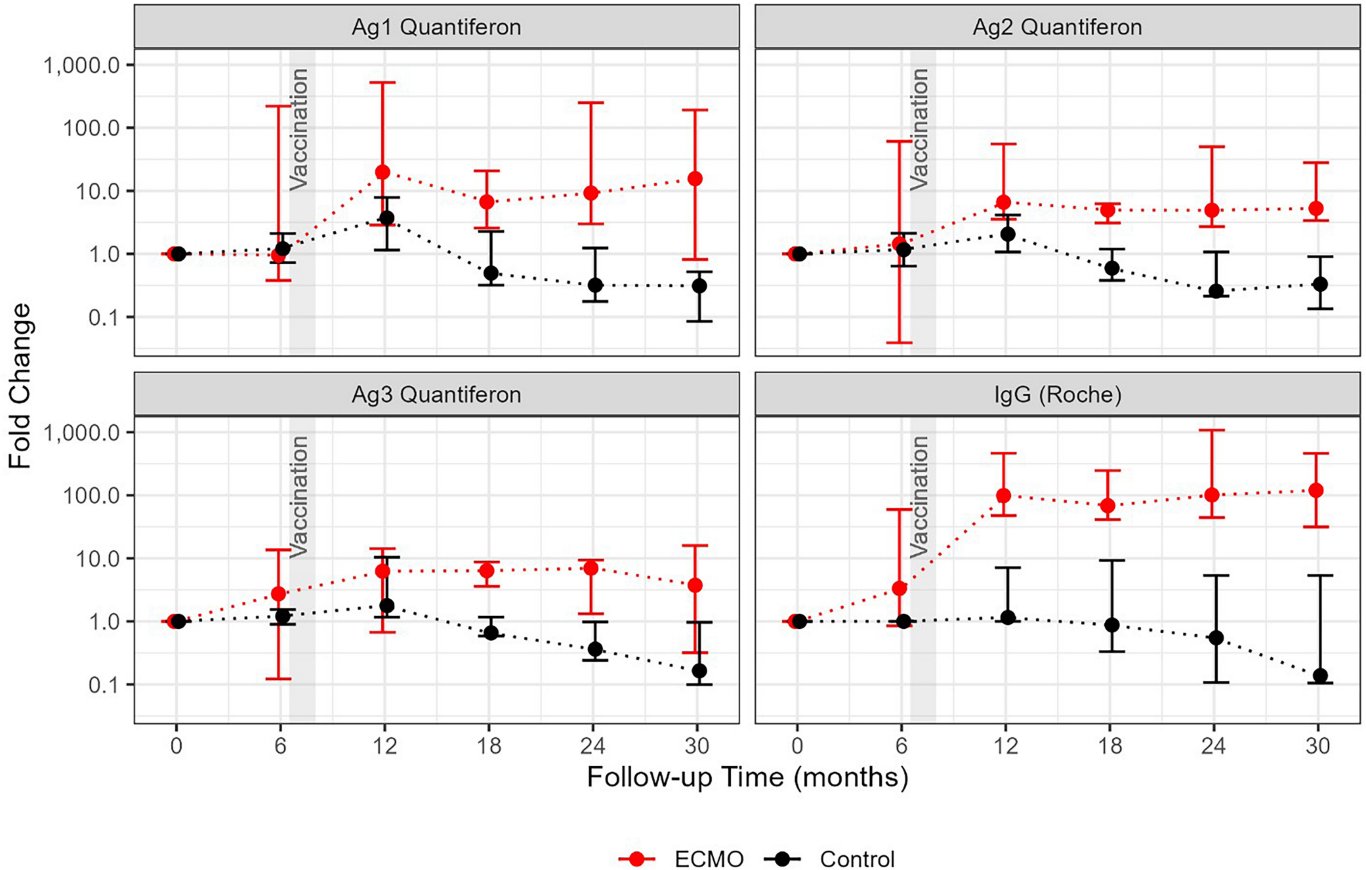
Dynamics of the anti-SARS-CoV-2 immune response in patients requiring ECMO compared to patients with less severe infection. Median fold changes compared to the initial values (the first available data point for each individual) at follow-up visits and the bootstrapped confidence intervals (95%) are shown as error bars. The colors correspond to the patient cohorts: patients requiring ECMO (in red) and the control group (in black).

At the end of the 6th month postinfection, all members of both groups received an RNA-based SARS-CoV-2 vaccine. Remarkably, the cellular immune response of the ECMO group was significantly stronger than that of the control group and was sustained after vaccination. Two years after COVID-19, both humoral and cellular immune responses were significantly greater in the ECMO group (Figure 2). The changes in the humoral and cellular immune responses of the two groups at each time point clearly showed an accelerated immune response over time in the ECMO group (Figure 3).

### 3.5 Psychology

By analyzing the correlation between the days spent in ECMO (mean ± SD: 15.9 ± 7.8) and the first measurement (M6) of the psychological questionnaire, we found a positive, significant correlation with depression (*r* = 0.727; *p* < 0.05), anxiety (*r* = 0.848; *p* < 0.01), and posttraumatic stress (*r* = 0.834; *p* < 0.01), although a negative, strong correlation was found for positive affectivity (*r* = −0.868; *p* < 0.01).

The comparative results of the psychological tests for the two data collection points are shown in Table 5. The mental health of the participants did not change significantly between the two measurements.

**TABLE 5:** Comparative analysis of the two measurements of the psychological test (M6–M24).

Variable	BDI	STAI	PDS	SL	PANAS positive	PANAS negative	SF-36 physical health	SF-36 mental health
*Z*	−0.705^a^	−0.981^b^	−0.780^b^	−1.089^a^	−1.423^a^	−0.070	−0.422^a^	−0.059^b^
*p*	0.481	0.326	0.436	0.436	0.155	0.944	0.673	0.953

^a^Based on negative ranks;

^b^based on positive ranks; Z = *z*-value. Paired-sample Wilcoxon signed rank tests. BDI, Beck Depression Inventory; STAI, Stait-Trait Anxiety Inventory; PDS, Posttraumatic Diagnostic Scale; SL, Satisfaction with Life; PANAS, Positive and Negative Affect Schedule; SF-36, 36-Item Short Form Health Survey.

The psychological test scores of each patient are presented in Table 6.

**TABLE 6:** Descriptive statistics.

Patients	BDI 1	BDI 2	PDS 1	PDS 2	SL1	SL 2	SF-36 physical 1	SF-36 physical 2	SF-36 mental 1	SF-36 mental 2
Patient 1	0	3	0	0	9	9	395	395	368	378
Patient 2	2	4	7	4	9	9	265	327.5	340	342
Patient 3	4	7	2	5	8	9	185	160	348.8	253.3
Patient 4	1	5	3	0	10	10	122	162.5	358	313.3
Patient 5	5	2	2	4	8	10	320	275	297.5	146.5
Patient 6	2	2	6	1	8	9	380	335	382	387
**Patient 7**	**19**	**17**	**24**	21	**2**	**6**	**202.5**	**247.5**	**136.8**	**171.8**
**Patient 8**	**10**	**5**	**23**	**36**	**9**	**9**	**102.5**	**52.5**	**203.8**	**167**
Patient 9	5	10	17	13	9	7	115	207.5	283	220.7

“1,” first data collection (M6); “2,” second data collection (M24); BDI, Beck Depression Inventory; PDS, Posttraumatic Diagnostic Scale; SL, Satisfaction with Life; SF-36, 36-Item Short Form Health Survey. In patients 7 and 8, mild to moderate levels of depression were observed. In these cases, a higher level of posttraumatic stress is present, consequently producing lower scores in physical and mental health. Life satisfaction was significantly worse for Patient 7 than for the other patients.

In two patients (Patient 7 and Patient 8) mild to moderate levels of depression were observed. In these cases, a higher level of posttraumatic stress is present, consequently producing lower scores in physical and mental health. Life satisfaction was significantly worse for Patient 7 than for the other patients. Although we did not find significant differences in the psychological test results, these results are likely due to the small sample size and calls for further research in this area.

### 3.6 Quality of life

Patients reported a significant improvement in their subjective health status at 24 months compared to at 6 months (subjective health status at the 6-month vs. the 24-month: median 50% vs. 80%, *p* < 0.01). However, the majority of patients continued to report a range of physical and psychological symptoms 24 months after their COVID-19 infection. Fatigue and a marked reduction in physical activity were particularly prominent (Table 7). Consistent with these findings, the average body mass index increased from 29 to 35 kg/m^2^, with 22% of patients classified as obese at the time of infection and 89% at the 24-month follow-up.

**TABLE 7:** Subjective health status of ECMO survivors.

Variable	Follow-up at 6th month	Follow-up at 24th month
Health status (%) median (IQR)	50 (40–90)	80 (60–95)
**Work**		
- No (n, %)	4 (44%)	3 (33%)
- Different (n, %)	2 (22%)	3 (33%)
- Same as before (n, %)	3 (33%)	3 (33%)
Sport (hours/week) median (IQR)	5 (2.5 to 6.25)	0 (0 to 2.25)
**Energy level**		
- Very low (n, %)	1 (11%)	0 (0%)
- Low (n, %)	2 (22%)	2 (22%)
- Medium (n, %)	6 (67%)	5 (56%)
- High (n, %)	0 (0%)	2 (22%)
**Fatigue**		
- Mild (n, %)	0 (0%)	2 (22%)
- Medium (n, %)	3 (33%)	4 (44%)
- Heavy (n, %)	6 (67%)	3 (33%)
Muscle pain (n, %)	5 (56%)	3 (33%)
Joint pain (n, %)	7 (78%)	7 (78%)
Palpitation (n, %)	9 (100%)	5 (56%)
Coughing (n, %)	4 (44%)	0 (0%)
Psychological consultation (n, %)	5 (56%)	3 (33%)
Physiotherapy (n, %)	9 (100%)	1 (11%)
**Tension**		
- Rare (n, %)	3 (33%)	7 (78%)
- Medium (n, %)	3 (33%)	0 (0%)
- Often (n, %)	3 (33%)	2 (22%)
Concentration problems (n, %)	9 (100%)	5 (56%)
Irritability (n, %)	6 (67%)	5 (56%)
Temper tantrum (n, %)	5 (56%)	5 (56%)
Impatience (n, %)	6 (67%)	6 (67%)
Mood instability (n, %)	6 (67%)	5 (56%)
**Sexual activity**		
- Asexuality (n, %)	2 (22%)	2 (22%)
- Strongly decreased (n, %)	2 (22%)	2 (22%)
- Decreased (n, %)	3 (33%)	3 (33%)
- Same as before (n, %)	2 (22%)	2 (22%)
Sleep disturbance (n, %)	4 (44%)	4 (44%)
Loss of hair (n, %)	5 (56%)	5 (56%)
Anosmia (n, %)	2 (22%)	2 (22%)
Ageusia (n, %)	2 (22%)	2 (22%)
Menstrual disorders (n, %)	3 (100% of female)	3 (100% of female)
Finger paresthesia (n, %)	2 (22%)	2 (22%)
**Limitation of life quality**		
- No (n, %)	0 (0%)	2 (22%)
- Mild (n, %)	2 (22%)	1 (11%)
- Moderate (n, %)	1 (11%)	1 (11%)
- Great (n, %)	6 (67%)	5 (56%)

The results of the QoL questionnaires assessed more than 2 years after acute infection are displayed in Table 8. Sleep-related questionnaires showed higher normal daytime sleepiness values for the ESS and elevated scores for the FSS, suggesting that the patients experienced fatigue and sleepiness in everyday life. The VAS score was less than the average Hungarian age- and sex-adjusted values for 5 patients. Overall, the evaluation of these questionnaires suggested that former ECMO patients still have impaired quality of life in some domains more than 2 years after contracting COVID-19.

**TABLE 8:** Results of the QoL VAS and sleep questionnaires > 2 years after COVID-19.

Variable	COVID-19 ECMO group (*N* = 9) [median; (range)]
Visual Analog Scale (VAS)	75 (30–90)
Epworth Sleepiness Scale (ESS)	8 (3–10)
Fatigue Severity Scale (FSS)	40 (19–58)

## 4 Discussion

We have found no significant organ damage behind the clinical symptoms frequently reported by our patients. In the recent literature, several studies have been conducted with the objective of evaluating the physical and mental status of patients who are affected by the acute phase of SARS-CoV-2 infection. Impaired pulmonary function and pulmonary-related symptom burden are frequently associated with postacute sequelae of coronavirus disease 2019 (PASC), which many patients with this disease experience after acute infection (23). This may have an impact on patients’ quality of life and general well-being. Pulmonary functionality can be evaluated objectively through PFTs, diffusion measurements, and 6MWTs. In accordance with the findings of other studies (24, 25), the PFTs of our patient population were almost normal, with only slight reductions in results and a restrictive pattern. It has been previously reported that CO diffusion capacity is impaired in patients with underlying residual fibrotic-like changes in the lungs, with some studies indicating that CO diffusion capacity does not return to normal at follow-up visits (24, 26, 27). In our study, patients who were able to undergo longitudinal measurements showed a notable steady improvement in DLCO and KLCO. DLCO (diffusion capacity) and 6MWD or exercise testing are often more sensitive to gas exchange impairment. Although standard PFT parameters did not change significantly, analysis of DLCO/6MWD values (where available) may provide important additional information. Based on this consideration, we recommend regular DLCO recording in long-term follow-up, as DLCO decline has been previously associated with radiological residuals, even when spirometry appeared “normal.”

During the COVID-19 pandemic, pulmonary radiomorphological features on chest CTs have been thoroughly studied; therefore, a typical progression pattern was observed during the course of the disease (28, 29). Chest CT imaging and assessment of the lung parenchyma played crucial roles in the diagnosis and follow-up of our study cohort. Considering that the patients included in this study suffered from particularly severe COVID-19 pneumonia, the findings of the baseline scans performed on admission showed severe and widespread pneumonia with predominantly peripheral ground-glass opacities (GGOs) and patchy consolidation patterns, corresponding to the known signs of the early active phase of the infection. It should be noted that in some patient cases, different stages of the disease could be observed on the same scan even at the 24-month evaluation. As a sign of disease progression, GGOs and consolidation patterns sometimes coexisted with varying degrees of residual subpleural banding on the baseline scan. A trend toward significant regression was observed at the 6- and 24-month follow-up CT scans. Nonetheless, residual fibrotic lesions were present on the majority of scans. In two patients, residual GGOs persisted on follow-up examinations (30). The observed presence of residual lung changes aligns with international results on this topic. However, although our cohort of patients was considerably smaller than the populations studied in more extensive international investigations, the prevalence of residual fibrosis appears to be relatively greater in the patients evaluated in our study (31). This may be attributed to the extreme severity of the illness in our study population. Most localized fibrotic bands (focal scarring) are clearly visible on radiological images, but do not necessarily cause measurable changes in standard global respiratory function tests (spirometry, TLC). Compensatory mechanisms (average performance of other, normal parts of the lung) maintain overall lung function. This is CT–PFT discordance, which has been described in several follow-up studies, and no close correlation has been found between quantitative CT parameters and PFT results (32, 33).

Following the severe COVID infection dominantly GGO was seen over a longer time, consistent with the slow resolution of alveolar-level injury. In regions where the insult was likely more pronounced, limited scar formation can be observed as a residual structural change, although pulmonary function remains preserved. The general improvement of the lung parenchyma was associated with significant improvement in the functional status, partly attributable to recovery of the respiratory pump, including improved diaphragmatic strength. The clinical significance of morphological radiological residuals (subjective symptoms, quality of life, progression) requires further, longer-term follow-up.

Cardiovascular diseases are known risk factors for severe COVID-19, and SARS-CoV-2 infection can also cause cardiovascular complications (e.g., heart failure, myocardial infarction, thromboembolism, myocardial injury, arrhythmias) (34). Initial data revealed an alarmingly high rate of cardiac MR abnormalities suggestive of myocarditis even after mild COVID-19 (35), but this high rate was not confirmed by subsequent studies (36–38). In the group of patients with severe COVID-19 detected in the present study, no patient had an increase in hs-troponin T levels suggestive of myocarditis, and their left and right ventricular functions were good both during acute infection and during follow-up. On echocardiography, patients had mildly dilated right ventricles in the acute period as a consequence of severe pulmonary injury (39, 40). Cardiac MR examinations during follow-up did not reveal contrast enhancement or mapping abnormalities suggestive of myocardial damage. A comparison of the cardiac MR parameters of COVID-19 patients and healthy controls revealed that COVID-19 patients had lower ventricular volumes, which based on our test results can be explained by the physical inactivity of the patients after ECMO treatment.

Our immunological findings demonstrated that patients infected with SARS-CoV-2 infection, whose condition had become sufficiently critical to require ECMO support were not only able to mount an immune response, but that this response was also sustained over the long term. This finding is also supported by the fact that the effect of the humoral and cellular immune response in the ECMO group was more pronounced than that in the control group in the long term. In the control group, the humoral and cellular immune responses declined after 12 months, whereas in the ECMO group, cellular immunity was consistently elevated, which was only partly explained by the vaccination. The immunological background of this requires further research.

Age has a significant impact on the dynamics of innate and adaptive immune responses (41). In older individuals, immunosenescence leads to a reduced pool of naive T cells, accumulation of senescent or terminally differentiated T cell subsets, impaired antigen presentation, and diminished cytotoxic responses (42). Such changes may impair effective viral clearance and the ability to control inflammation. Simultaneously, inflammaging may prime the immune system to mount a dysregulated, over-exuberant inflammatory response (“cytokine storm”) upon infection or other insults (43). In contrast, younger, immunocompetent individuals may mount a more balanced but robust immune response, capable of producing a more explosive pro-inflammatory response under certain circumstances. For example, studies in COVID-19 patients have shown that age influences immune cell subset distributions and cytokine profiles: younger patients may have different patterns of T cell activation, exhaustion, and cytokine production compared to older patients (44). While many existing data emphasize that older age is a risk factor for severe disease, the immunological picture is complex, and in some younger patients, hyperinflammatory responses may also contribute to more severe tissue injury (45, 46). Several autopsy studies report that certain clinical conditions (e.g., severe COVID-19) younger patients show different, sometimes more pronounced organ/histological changes, which supports the idea that there may be age-related differences in tissue damage (47). Our study population was relatively young (mean age ∼34 years), which may have influenced both clinical presentation and long-term outcomes. One potential immunological explanation for our observations may be related to age-specific differences in immune response dynamics. In our cohort, the relatively young age might have allowed for a sufficiently strong and rapid immune response that cleared the insult efficiently, but at the same time induced localized tissue changes (e.g., fibrotic remodeling) through inflammatory processes, without resulting in sustained systemic immune exhaustion. Nevertheless, we acknowledge that mechanistic confirmation of this hypothesis requires further dedicated studies. While we primarily focused on clinical, radiological, and functional outcomes, future directions should include standardized immunological assays, such as flow cytometry for T and B lymphocyte subsets (naïve, memory, exhausted), activation and co-stimulatory markers, and serum cytokine profiling (e.g., IL-6, IL-1β, TNF-α, IL-10). These would allow correlation of immunophenotype with clinical and morphological outcomes and help to elucidate whether localized fibrotic changes relate more to direct viral injury or to immune-mediated damage.

Experiencing critical illness and undergoing ECMO therapy can impose a substantial psychological burden. A Canadian population-level cohort study examined the incidence of new mental health diagnoses among ECMO survivors compared to that among ICU survivors without ECMO treatment (48). The non-ECMO survivors had a 14.5 per 100 person-years incidence of new psychiatric diagnosis, while the ECMO survivors had 22.1 per year, which means that they are at increased risk (24%) of developing mental health problems. The most commonly reported mental health conditions (in both groups) were mood disorders, anxiety disorders and posttraumatic stress disorder (PTSD). A review in 2020 examined the cognitive, psychiatric and quality-of-life outcomes of ECMO treatment among survivors, with a median follow-up of 36 months (49). The most common psychiatric outcomes were depression, anxiety and PTSD. The prevalence of depression among the included studies reached 43%, the prevalence of anxiety ranged from 15 to 50%, and the prevalence of PTSD reached 41%. Regarding quality of life, the physical quality of life of ECMO survivors was worse than that of the standard population, although it seemed to improve over time. The mental quality of life did not differ significantly from the population norms. Knudson et al. (7) analyzed 31 studies examining the health-related quality of life and psychiatric symptoms of ECMO survivors. The authors found that physical quality of life was worse than mental quality of life, and a significant portion of the included patients reported anxiety, depression and PTSD symptoms (7). In our present study, we found significant associations between the duration of ECMO treatment and symptoms of depression, anxiety, and PTSD, indicating that longer periods of ECMO were linked to greater levels of psychological distress. This aligns with the findings of the previous studies discussed above. Overall, these findings suggest that ECMO survivors may face ongoing mental health challenges after treatment. This highlights the need for continued monitoring and support for ECMO patients, as well as interventions aimed at addressing their psychological needs. Additionally, the study underscores the importance of further research to better understand the long-term psychological effects of ECMO support.

Patients’ quality of life declined temporarily following ECMO support; however, by the second year of follow-up, most had returned to work, though with some limitations in their daily routine. Furthermore, no definite permanent organ or functional impairment was identified to explain the higher rates of subjective complaints (fatigue, palpitation, effort dyspnea). Our findings suggest that these symptoms are not directly related to the viral infection itself but rather represent sequelae of ICU treatment. This appears to be partly of psychological origin (depression, anxiety, posttraumatic stress), which led to lifestyle changes. During follow-up, the BMI of ECMO treated patients—already elevated at the onset of COVID-19 infection—increased significantly and by the end of the second year of follow-up, 88.9% were classified as morbidly obese. The poorer 6MWT results were primarily attributable to obesity, lack of stamina and physical inactivity rather than pulmonary or cardiac limitations. Recognizing this, we emphasize the importance of initiating exercise rehabilitation and psychological support early in the ICU, and maintaining them over the long term (50). At the same time, the relatively young age of our cohort (mean ∼34 years) may represent a potential source of selection bias, as younger individuals generally have fewer chronic comorbidities, better baseline cardiopulmonary reserve, and greater regenerative capacity. This may have contributed to the faster and more complete cardio-pulmonary recovery observed in our study compared with older or multimorbid populations. Larger studies across broader age ranges are required to confirm whether our findings apply more generally.

## 5 Limitations

Our findings are subject to several limitations. First, limited sample size: this study was conducted at a single-center and involved a small sample size. The follow-up included only 9 ECMO-treated patients, which limited statistical power and generalizability. Second, lack of pretreatment baseline conditions: baseline (pre-treatment) data were unavailable, preventing us from attributing all observed changes solely to COVID-19 or ECMO treatment. Third, due to the heavy burden of the COVID-19 pandemic on the healthcare system, some data were missing at discharge (e.g., 6-month pulmonary function test results). Fourth, this cohort represents an unique group of ECMO survivors, making it impossible to select one appropriately matched control group; therefore, separate control groups were used for cardiological and immunological comparisons. A further limitation of our study is the relatively young age of the cohort (mean ∼34 years). This demographic may not reflect the typical patient population with chronic comorbidities or age-related immunological changes (immunosenescence, inflammaging), and therefore our findings may not be directly generalizable to older or multimorbid populations. Moreover, we did not perform komplex immunophenotyping (e.g., T-cell subsets, cytokine panels) which limits our ability to draw mechanistic conclusions regarding the role of age-dependent immune responses.

## 6 Conclusion

In conclusion, all our patients who survived the acute phase of COVID-19 disease with ARDS requiring ECMO treatment survived at least 2 years of follow-up. Remarkably, at the end of this period, all our patients still demonstrated a sustained and robust immune response. ECMO support in patients with ARDS from appropriately selected severe COVID-19 disease may result in favorable functional outcomes. Two years after recovery from infection we observed no lasting heart or lung damage. However, a significant proportion of patients still have psychological and musculoskeletal problems that affect their quality of life. Some of these problems are the result of COVID-19 disease and the associated ARDS, and some resulting of long ICU stays.

## Data Availability

The original contributions presented in this study are included in this article/supplementary material, further inquiries can be directed to the corresponding author.
